# New Multilevel Partnerships and Policy Perspectives on Active Ageing in Italy: A National Plan of Action

**DOI:** 10.3390/ijerph17249585

**Published:** 2020-12-21

**Authors:** Francesco Barbabella, Eralba Cela, Claudia Di Matteo, Marco Socci, Giovanni Lamura, Pietro Checcucci, Andrea Principi

**Affiliations:** 1 Centre for Socio-Economic Research on Ageing, National Institute of Health and Science on Ageing (IRCCS INRCA), 60124 Ancona, Italy; f.barbabella@inrca.it (F.B.); eralba.cela@unimi.it (E.C.); claudia.di_matteo@soch.lu.se (C.D.M.); g.lamura@inrca.it (G.L.); a.principi@inrca.it (A.P.); 2 Department of Social and Political Sciences, University of Milan, 20122 Milan, Italy; 3 National Institute for Public Policy Analysis (INAPP), 00198 Rome, Italy; p.checcucci@inapp.org

**Keywords:** active ageing, older people, social policy, social participation, social inclusion, Italy, COVID-19

## Abstract

Active ageing (AA) policies aim to improve quality of life of older people by enabling better social participation and inclusion. Despite many international initiatives to promote AA undertaken in recent years, Italy did not systematically address this policy challenge until very recently. This paper presents the first national Plan-of-Action (PoA) (2019–2022) adopted by this country for supporting policy design and recommendation in this field. The PoA aims to create a multilevel, co-managed coordination of AA policies, by involving a network of national and regional policy makers, experts, researchers and stakeholders in civil society. The ad-hoc consultation process established for this purpose helps the recognition of different interests and expectations on AA, fostering new solutions by involvement, consultation and joint discussion of policy options. The PoA is designed to cover the traditional policy cycle, including the stages of agenda setting, policy formulation, decision-making, implementation and monitoring. At the end of the period covered by the PoA, an Italian AA Strategy will be launched to achieve systematic impact in this field, thus ensuring a long-term, sustainable impact on national and regional policy makers, civil society and research community.

## 1. Introduction

Population ageing is a longstanding process transforming the demographic, social and economic facets of societies worldwide, especially in the European Union (EU) [1,2] where the old-age dependency ratio reached 31.8 in 2019 against 24.9 in the United States [3]. It is estimated that the segment of people aged 65 and older will grow from 19.7% to 29.5% of the total EU population between 2018 and 2060, with an even greater increase of people over 80 years, who will double (from 6% to 12%) in the same period [4]. Likewise, the EU population in working age (15–64 years) will decline from 65.2% to 56.1% over 2016–2060 [1], affecting the labour market composition and societies as a whole.

At the European level, the concept of active ageing (AA) has been used in the last decades by policy makers, intergovernmental organizations and the research community for defining and promoting new perspectives on ageing individuals and later life, rejecting the idea of considering older people as just care receivers or passive citizens [5,6,7,8]. According to the World Health Organization (WHO) [7] (p. 12), “Active ageing is the process of optimizing opportunities for health, participation and security to enhance quality of life as people age”. Similarly, the EU acknowledged that the aim of an AA policy approach is to boost the potential of older people, by optimizing opportunities for physical, social and mental well-being throughout the life course, which should lead to “a high quality of life for people of all ages, improved productivity and a move towards strong solidarity between generations in our ageing societies” [9] (p. 7).

The concept of AA, as defined by the WHO [7], is based on three pillars: participation, health and security. The individual capacity for AA depends on eight groups of determinants, related to physical environment, health and social services, behavioural (healthy behaviours, addictions, medications), personal (biology, genetics and psychology), social (social support, violence and abuse, education and literacy) and economic factors (income, social protection, work), as well as gender and culture [7]. The policy response should aim to mitigate barriers and risk factors, empower individuals during the life-course and enable opportunities for AA. Generally speaking, AA refers to a broad range of activities enabling ageing individuals to participate in and contribute to society to the largest extent [5,10,11,12], impacting positively not only on active individuals–at micro-level, on their quality of life (QoL) and physical, mental and social well-being—but also on the society as a whole—at macro-level, on social cohesion, welfare systems and public expenditure [6,13].

However, debates around AA produced some criticism against the concept and policies inspired by it [14]. Critics pointed out that AA policies and initiatives might lead to disruptions like the application of a normative approach towards ageing; the neglect of the possibility of achieving AA if a person experiences physical or mental health problems; and the emphasis of a productive model of ageing, based on older people’s contribution to society (e.g., employment). Similar concerns have been raised also in relation to policy tools like the Active Ageing Index (AAI) [15], developed by the European Commission and the United Nations Economic Commission for Europe (UNECE) to monitor and identify AA policy intervention areas across countries [16]. Furthermore, in a first phase (until late 1990s–early 2000s), AA was mostly used for referring to a specific set of policies, designed and implemented to respond to socio-economic challenges related to demographic trends, i.e., increase of retired people and rise of financial expenditure for pensions. In this sense, AA potential was limited in practice to achieve the policy goals of increasing employment rates in later life, reducing early retirement and increasing retirement age, aiming to a general extension of working lives [14,17]. This de facto narrowed the AA scope and reduced it to the purpose of achieving higher employment rate for older workers, without focusing on other important dimensions.

Nonetheless, in the last twenty years, policy makers and academic communities have been making important steps forward. More and more initiatives at international, national and regional levels have been conducted to clarify the multidimensional nature of AA and adopt coherent, integrated policies for addressing needs, attitudes and interests of ageing individuals.

At international level, important initiatives occurred. Among them, the following played a major role: the approval of the Madrid International Plan of Action on Ageing (MIPAA) at the Second World Assembly on Ageing of the United Nations (UN) in 2002 [18]; the UNECE definition of a regional strategy for the European Region (so-called MIPAA/RIS) in 2002 [19]; the organization of the European Year of Active Ageing and Solidarity between Generations in 2012 [5]; the adoption of the AAI at the 3rd Ministerial Conference on Ageing organized by the UNECE in 2012; the commitment to the UN 2030 Agenda and its Sustainable Development Goals (SDGs) by member states of the 4th UNECE Ministerial Conference on Ageing in 2017; and the acknowledgment of the close relation between population ageing and economic, social and environmental development [20].

Furthermore, several policy initiatives took place during last decade, at national or subnational levels, especially in Europe, with the purpose of adapting welfare arrangements with the AA paradigm or providing innovative policy frameworks which are at least in part consistent with or inspired by it. Recent examples can be represented, without claiming to be exhaustive, by the National Strategic Policy for Active Ageing of Malta 2014–2020 [21], the Active Ageing Strategy of the Republic of Slovenia [22], the National Action Plan Promoting Positive Ageing for 2013–2017 in the Czech Republic [23], Austria’s Federal Plan for Senior Citizens: “Ageing and the Future” [24], Ireland’s National Positive Ageing Strategy [25], the German National Demographic Strategy: “Every Age Counts” [26]; Bulgaria’s National Strategy for Active Ageing [27], and the Government strategy to enhance Age-friendly communities in Canada [28].

Within this context, Italy represents an interesting case study, given the highest proportion of persons aged 65 years and over (22.8% in 2019) in Europe and the presence of a limited toolbox of AA policies. Although the Italian government contributes to international programmes focused on ageing and AA (like MIPAA) and there is an increasing interest to monitor AA [29,30,31], no national policy addresses older people and ageing in line with AA concept and framework, despite some sporadic attempts for new framework laws on AA (which never reached the voting stage in the national Parliament). Some initiatives exist at regional level, but these often present drawbacks on the implementation side, for instance in terms of few resource allocation, which jeopardize the effectiveness of the undertaken policy efforts [13].

In principle, national policies designed specifically for older people are limited, since the Italian welfare system is still predominantly familistic [32], i.e., families are assigned de facto most responsibilities for sustaining and caring for their members. Formally, public policies tend towards a universalistic approach, without a rigid division between age groups and aimed to address the general population. Comprehensive definitions of older people or AA do not exist in national policies. AA is sometimes mentioned in relation to national health plans (as an approach stimulating active and healthy ageing of individuals), whereas older people are the explicit target group of few policy measures (e.g., old-age pensions, home help for older people by municipalities).

The role of social policies is mostly distributive by means of cash-for-care schemes (pensions, care allowances, etc.), so that older people are usually categorised as a frail target group. The implicit national policy discourse that emerges lead to consider ageing either as an issue concerning long-term care (i.e., viewing older people primarily, if not exclusively, as care recipients) or as a matter of labour market participation and public spending for pensions (i.e., prompting a higher increasing retirement age to improve sustainability of the pension system).

Concerning participation in the labour market, an evident conflict exists in policy discourses between, on the one hand, the need to retain older people at work (by extending working life and postponing retirement age, mostly due to maintaining sustainability of the pension system) and, on the other hand, the need of ensuring to (some or most) older workers the availability of appropriate working conditions and employability [33] in light of prolonged working lives (also, among other possible initiatives, by decreasing retirement age for some groups and implementing other pre-retirement measures). Such policies are based on a limited dualist view of old age. On the one hand, older people are asked to contribute as much as possible to the economy and financial sustainability of the welfare system, by remaining longer at work. On the other hand, retired people are mostly seen as passive recipients of pensions and care services.

These facts confirm that the public discourse about population ageing still depicts older people with characteristics such as frailty and lack of autonomy, instead of looking at them as active citizens and a resource for the community. Such a perception of ageing comes from the traditional approach of the Italian welfare system, which is still heavily influenced by an organisation based on social categories [34], reinforced by a marked concentration of public spending on the public pension system [35].

In general terms, the first policy attempt to introduce a nation-wide initiative on AA in Italy has been carried out only since 2019. The national government and the National Institute of Health and Science on Ageing (IRCCS INRCA) launched a 3-year Plan-of-Action (PoA) to fill this gap. The PoA aims to create a national, multilevel, co-managed coordination of AA policies by involving national, regional and local stakeholders. Its ultimate goal is to initiate an Italian Active Ageing Strategy (thereafter IAAS) in 2022, which could sustain a well-structured network for constantly exploiting and transferring good practices, producing further AA policy/practice developments and interacting with international bodies and other governments active in this field. For this purpose, the EU policy framework and the programmatic documents developed internationally [7,12,19] offered a helpful guidance in preparing the conceptualization and implementation of AA policies in Italy.

The aim of the paper is to describe this recent policy innovation in the field of AA in Italy, i.e., the building of a research-driven PoA for improving policymaking on AA at regional and national levels.

## 2. Methods

The PoA adopts a policy analysis perspective for a proactive policy design and recommendation based on an interactive style [36]. In particular, the ultimate aim is to reformulate and translate knowledge about the Italian policy framework into new tailored national and regional AA policies. In this respect, the PoA constitutes a policy innovation [37,38] which benefits from Evidence-Based Policy Making (EBPM) [39] activities and an active engagement of relevant actors [40].

According to Mayer et al. [36], a policy analysis targeting “design and recommend” activities involves “generating a set of alternative strategies that each consists of several tactics aimed at achieving particular objectives or sub-goals” (p. 7). Researchers are indeed supportive to policy makers “by advising or by making (partial) policy designs in terms of ‘actions–means–ends’” (p. 7). In this sense, we aimed to proactively design a wide process (i.e., the PoA) of policy design for supporting national and regional policy makers. The key feature of the designed process relies on an “interactive style” [36], which “assumes that individuals–experts, analysts, clients, stakeholders and target groups–have or may have differing views of the ‘same’ policy problem” (p. 14). Furthermore, “target groups and stakeholders are usually invited to structure problems or devise solutions in structured working meetings” and “this brings about a multiple interaction whereby the views and insights of the analyst, the client and also the participants are enriched” (p. 14).

Based on the model of policy cycle [41], we designed the PoA as a 3-year process in order to (1) advance the knowledge about the current status of AA policies, (2) produce an evidence-based set of policy recommendations, and (3) advice and monitor (regional and national) policy makers in adjusting their AA policies. The process was elaborated with the purpose to suggest policy aims and link them instrumentally to new policy tools [42]. Indeed, we built a work plan for designing a policy innovation, which embraces complexity, is proactive and focused on citizens, shapes new alliances, facilitates stewardship, and emphasises impact. The work plan includes a participatory, interactive approach amongst national and regional policy makers, experts, researchers and stakeholders in civil society, in line with Edelenbos [40]. This approach helps the recognition of different interests and expectations on a policy issue, fostering new solutions by involvement, consultation and joint discussion of policy options. The work plan of the PoA was designed to cover the traditional policy cycle, including the stages of agenda setting, policy formulation, decision-making, implementation and monitoring [41].

In this regard, the PoA represents an action tool which exploits an EBPM approach [39] to support policy makers in national and regional governments for improving AA policy by means of coordination, systematisation and engagement of all key actors.

The concept of AA adopted in the study was retrieved from WHO [7] and concerned the ensemble of labour, social, educational and entertaining activities performed by older people (65+ years), bearing in mind that older adults are a heterogeneous group with different profiles, needs, resources and expectations. This broad definition identifies, amongst others, activities of social participation, education and training, work, culture, tourism, sport, leisure, informal care (e.g., elder care, grandparenting), social farming and gardening, civil engagement and volunteering, co-housing, and others. Against a possible misinterpretation of AA, in our study, we refer explicitly to AA as the opportunity for all ageing individuals to engage, access and use resources for satisfying own interests, attitudes and desires, despite any health, social or economic barrier they might face. Such a conceptual definition shall be intended as definitely inclusive and focused on individual agency and not on a normative or model ageing, overcoming most common critics to AA [14].

The team of researchers was composed by experienced staff at two Italian research institutions, i.e., the IRCCS INRCA and the National Institute for Public Policy Analysis (INAPP). The team remained the ultimate responsible for both the study and definition of the PoA. The funding agency—the Department for Family Policies (DFP), a governmental office of the Italian Presidency of the Council of Ministers—granted financial resources and collaboration to discuss the PoA, organise plenary meetings with stakeholders, and facilitate the access to other public organisations and relevant documentation. The whole research work was conducted in an ethical, transparent and accountable way, with all produced documents and protocols publicly available in the project website [43] and informed consents prepared and administered to each interviewed person (during the field research for the state of the art—agenda setting—of the PoA).

## 3. Results: A Plan-of-Action for Advancing Active Ageing Policies in Italy

### 3.1. Conception of the Plan-of-Action

The opportunity to give momentum to the discussion around AA at national level in Italy came firstly when, in May 2016, the World Health Assembly approved the Global Strategy and Action Plan on Ageing and Health 2016–2020 [44]. The Strategy, encouraging the adoption of a transformative approach to global ageing, streamlined its objectives with the Sustainable Development Strategy (Agenda 2030), already adopted by the UN General Assembly on 25 September 2015 [45,46], aiming at giving older people the capacity to face the complex changes occurring in their social and physical environment. The Strategy 2016–2020, which would later lead to the Decade of Healthy Ageing 2020–2030, aimed at mirroring what had been established in Agenda 2030.

In this context, in December 2018, an agreement was signed between the DFP and the IRCCS INRCA, a public national organisation that aims to conduct interdisciplinary gerontological and geriatrics research, contributing to a more holistic understanding of the ageing process. The agreement concerned a 3-year PoA project, which is also supported by both the Ministry of Labour and Social Policies and the INAPP, in their role of National Focal Point in Italy of the MIPAA/RIS implementation and monitoring. The project aimed to lay the foundations for the IAAS, by supporting Ministries, Regions and Autonomous Provinces (APs) in introducing new or more comprehensive and coordinated policy instruments tackling AA challenges.

The specific objectives of the PoA are the following:

1. To build and consolidate a national coordinated permanent multilevel consultation process with relevant stakeholders, to facilitate the promotion, networking and exchange of successful experiences in the area of policies and interventions on AA;
2. To propose and discuss with Ministries, Regions and APs shared approaches and methods for policies and interventions, providing support in their designing and implementation and monitoring their impact;
3. To elaborate guidelines for promoting policies, actions and interventions on AA and its role for improving social inclusion of older people and strengthening intergenerational solidarity;
4. To support the implementation of the MIPAA in Italy.

Specifically in relation to the latter objective, the PoA addresses and contributes to the achievement of international policy goals linked to ageing, including the 10 MIPAA commitments [19] (Mainstreaming ageing; Integration and participation; Economic growth; Social security; Labour markets; Lifelong learning; Quality of life, independent living and health; Gender equality; Support to families providing care; Regional co-operation) and nine out of 17 SDGs of the 2030 Agenda for Sustainable Development (No poverty; Good health and well-being; Quality Education; Gender Equality; Decent work and economic growth; Reduced inequalities; Sustainable cities and communities; Peace, justice and strong institutions; Partnerships for the goals), set in 2015 [46] and connected to the MIPAA [47]. The PoA incorporates the MIPAA and SDGs by adopting their commitments/goals as policy dimensions to analyse. Furthermore, the PoA is aligned with the Italian National Sustainable Development Strategy (NSDS) 2017–2030, enforced on 22 December 2017.

The PoA is the first initiative undertaken in this country at the national policy level for addressing specifically the ageing challenge and the potential of AA. Although the concept of AA dates back in early 2000s, as mentioned earlier, in Italy no policy attention has been given to it so far at the national level [48].

The main strength of the PoA is that it is based on a fully multilevel and participatory approach of national and regional institutional actors and other stakeholders. A continuous consultation process was initiated in 2019 and will proceed, with the strategic intention of maintaining the network in the long-term. The expectation is that all the actors involved could benefit from this unique coordinated opportunity of having national and regional governmental officers, policy analysts, representatives from the civil society, unions and academia engaged in assessing together the progress in AA and suggesting concrete actions to advance, if and when this is deemed to be necessary or useful.

In the following sections, we describe the PoA, its activities and expected outcomes, structuring them by stages of the policy cycle.

### 3.2. Work Plan and Activities

The PoA is aligned with MIPAA’s commitments and SDGs also in terms of methodology adopted. In fact, the core pillar of the PoA lies in the systematic involvement of all relevant national and regional stakeholders. In this regard, three main groups of stakeholders have been identified and involved in the stakeholder group since mid-2019 (Table 1): representatives of national government’s bodies (Ministries) and other relevant national public institutions (group A); representatives of all Regions/APs (19 Regions and 2 APs) who work (directly or indirectly) on AA policies and act as main interfaces with other regional staff members (group B); representatives of major national civil society organizations and federations, pensioners’ unions, academia, and other stakeholders (group C). One or more representatives were identified per each stakeholder and joined the stakeholder group.

With the support of the network of stakeholders created along the lines outlined above, during the project four sequential tasks will be conducted, following an iterative consultation process of all stakeholders. The four tasks include:

1. An analysis of the state of the art concerning AA policies in all 19 Italian Regions and 2 APs, and in Ministries (duration: 12–14 months);
2. The development of national guidelines to develop AA policies according to MIPAA’s principles and SDGs (duration: 5–7 months);
3. The definition of intervention areas in each Region/AP and at the national level, by using the guidelines produced, according to strengths and weaknesses highlighted by the previously carried out state of the art analysis (duration: 7–9 months);
4. The implementation and monitoring of regional interventions in the areas identified, also by means of AAI regional analyses (duration: 6–8 months).

These tasks will be made possible by the continuous, iterative and close consultation with and among all national and regional institutional actors (groups A and B) and the other stakeholders (group C), by means of face-to-face plenary meetings, surveys, individual interviews, focus groups, teleconferences and feedback to written documents and reports. The network of stakeholders will be involved in an iterative way during all four sequential tasks, by supporting the evaluation of the work carried out in each task and providing inputs about the work to be performed in the following task. The network meets in plenary (face-to-face or via videoconferencing) more times during the 3-year period, i.e., at the beginning and at the end of the first task, at the end of the project, as well as, possibly in the transitions between tasks, for discussing the results and defining the future IAAS.

### 3.3. Kick-off Event

At the beginning of PoA implementation (April–June 2019), the project team addressed the stakeholders by explaining the project’s purpose and the added value of a wide networking collaboration. A brief project presentation had been preliminarily circulated to them, together with invitations to attending the first planned plenary meeting in Rome, held on 24 June 2019. To promote the project, its PoA was also presented previously in other two occasions, i.e., at the Italian Forum of Public Administration (session on “Active ageing between demographic crisis and inclusive growth”, Rome, 16 May 2019) and at a meeting of the Technical Commission for Social Policies of the Conference of Presidents of the Regions (Rome, 5 June 2019).

The kick-off plenary meeting was attended by 51 representatives of the three groups of stakeholders (in some cases, there were more than one representative per stakeholder). The project staff presented the project, its tasks and tools in detail, as well as the role of the project team members and the expected tasks of the main representatives at each Region/AP and of other stakeholders. Furthermore, a 2 h session was dedicated to three parallel working groups, in order to obtain a feedback to refine and agree the proposed approaches and tools for conducting the analysis of the state of the art (i.e., the first step of the project) concerning the topic of AA policies. The active participation of attendees provided useful insights and concrete suggestions to improve the contents of methodological tools such as guidelines for interviews, among other documents.

### 3.4. First Task—Agenda Setting: The State of the Art

The aim of this first task was to analyse current laws, policies and interventions impacting (directly or indirectly) on AA at national and regional level. This has been done by means of case reports, which provided a thoughtful policy analysis and evaluation of current state of the art about AA in Italy. Results updated a previous attempt of systematic analysis and evaluation conducted in 2016 [49]. The project team, composed by seven experienced researchers with multidisciplinary background and experts in qualitative research, interacted with a designated main representative at each public institution (stakeholders in groups A and B). This latter was the key provider of information and acted as interface between researchers and other officers in the institution who work on preparing, implementing and monitoring policy initiatives on AA related issues. Researchers collected primary and secondary data in order to carry out the following tasks:

1. A preliminary secondary data analysis, based on information collected in terms of laws, policies, statistics and any other relevant data concerning AA;
2. A case study for each Region/AP and national governmental body, with planned interviews and focus groups with the main representative on AA and managers/officers in charge of supervising the selected relevant policies. Standard topic guides for interviews/focus groups and templates for reporting were used by researchers for conducting the field research (face-to-face or phone), as well as for reporting the results;
3. A more in-depth analysis of the state of the art in each Region/AP and at the national level, taking into consideration also economic, geographical, social and cultural differences.

National and regional reports were produced in 2020 [50] by using standard templates and made publicly available online [43]. They summarised results and provide insights about how to maintain and improve policies on AA.

### 3.5. Second Task—Policy Formulation: National Guidelines about Active Ageing Policies

On the basis of the state-of-the-art analysis, the project team initiated the second task with a plenary meeting with the network of stakeholders on 21 October 2020 for discussing the results obtained in the previous task and receiving inputs. The consultation will continue and enable the definition of common principles to be proposed and agreed by the national and regional stakeholders, in order to design a coordinated, coherent and comprehensive framework for social policies targeting older people and fostering their social participation and inclusion. The aim of this activity will be to identify:

1. Common principles, values and tools that can be shared across the national territory, also with reference to the current national legislative framework concerning AA and the division of competences between national and regional governments;
2. Recommendations for policy making in the field of AA, including best practices identified during the previous phase on the analysis of the state of the art;
3. For each Region/AP and at the national level, possible strengths and weaknesses in this policy area;
4. Contents of possible new national and regional laws on AA (or amendments to existing ones), which could support the systematisation of policies in the field, increase stakeholder engagement and foresee funding for designed measures.

The National Guidelines for AA policies emerging from the process described above will constitute a new document reporting all these aspects, to be finalized in collaboration with the network of stakeholders, in order to validate and ensure the widest and more inclusive approval of such general framework.

### 3.6. Third Task—Decision-Making: Identification of Intervention Areas

This task concerns the creation of an AA policy action plan in each Region/AP and at the national level, based on the results of the state-of-the-art analysis. The plan will apply the National Guidelines produced in the previous task in a flexible way (according to territorial specificities), enabling the regional and national governments to adapt to discuss and re-think the implementation of AA policies depending on the territorial needs, while respecting the statutory competences. The project team and national government will promote the importance of AA policies, thus making recommendations and supporting regional policy makers. The participatory approach applied since the beginning of the project shall help the establishment of a trustworthy and efficient collaboration between project team and regional institutions, thus facilitating the chances for mutual engagement and learning. If not already existing, in each Region/AP, the project will promote an interaction between regional government (politicians, managers, officers) and relevant stakeholders of civil society. Indeed, the aim of this task will be to co-define, together with regional institutions and stakeholders, a concrete set of priorities in terms of policy areas and policy tools to implement, for advancing and improving AA policies.

Some Regions/APs are already advanced, with proper general laws on AA or on some of its key dimensions (e.g., volunteering, caregiving, cultural and social participation, labour market, etc.) and efficient instruments for implementation and monitoring. In these cases, the discussion will focus on whether and possibly how to improve the status and the suitability and modalities of transferring these models to other Regions/APs. In those Regions where only fragmentary policies or no specific laws exist, a cultural change (within the institution and across society) and/or the approval of useful policy tools will be proposed. Communication and awareness campaigns will be developed as well for promoting a deep change in the social recognition of older people and of their rights and opportunities for a complete and satisfactory social participation.

With regard to this task, the project team will act as advisor for regional actors, by supporting the definition of the policy issues, the interaction with the civil society for consultation and co-decision, the decision-making process, and the shaping of actual (legislative and non-legislative) tools.

### 3.7. Fourth Task—Implementation and Monitoring: Follow up of National and Regional Policies

The last task regards the follow-up of the status and the progress of the implementation of policy strategies in the AA area, as planned in the previous task. On the basis of their starting level (e.g., no law on AA, fragmentary laws, single comprehensive law, and whether and how these policy tools are actually used), Ministries and Regions/APs will be approached and supported by the project team also for gaining further data about the implementation of AA policies. The project team will collect available secondary data (from regional main representatives and statistical data series), as well as primary data (with further site visits, when necessary). These data will enable researchers to update the policy analysis for each Region/AP and conduct a final evaluation of policy design and implementation, in order to conclude the PoA phase and define and launch the IAAS for the next years.

Throughout the project, the AAI will be also used for complementing qualitative information with quantitative data, to check possible gaps and so providing further inputs for policy making. This effort will better align Italy with the European context, since the AAI is already employed by European Commission and UNECE as a statistical tool for analysing the EU policy responses to the population ageing challenges [51] (The AAI is a composite index–the higher the score, the higher level of AA is reached by the country–and is based on 22 indicators, which synthesize AA across four different domains: employment; social participation; independent, healthy and secure living; capacity and enabling environment [16]). Some EU Member States already took an active role and use the AAI for policy purposes [48,51,52,53,54].

## 4. Discussion

Despite the rising interest in AA by the government and stakeholders in civil society, ageing is still a topic mostly framed between long-term care and pension system in the Italian context. That is, older people are conceived by policies prevalently as passive receivers of services (e.g., care) or benefits (e.g., pensions). In this respect, the Italian policy framework suffers from lack of recognition of older individuals’ actual or potential active role for formal and informal activities (e.g., volunteering, informal care); disinterest in addressing new ways of engagement, including leisure, consumption and personal empowerment (e.g., travels, training), considering expectations and motivations of older people; neglect of the life-course approach and the importance of supporting AA across all life stages. Moreover, despite the adoption of a “health-in-all policies approach”, inspired by WHO suggestions, Italy did not yet take firmly the road to the holistic approach that should be at the root of AA. Current inequalities among older people, as depicted for instance by the implementation of AAI in Italy [48], suggest that the traditional rigid representation of the life-course does not appear to be sustainable anymore. This is particularly evident as we look at the complex relationships which link together professional career and the need for life-long learning, or lifestyles and the early onset of functional limitations and disabilities [55].

This paper presented the PoA as an EBPM innovation that will lead to new AA policy design and recommendations to address these gaps within the Italian context. The PoA represents a unique opportunity to promote the development of a national AA strategy in Italy, which has been absent in the policy discourse so far. The PoA is a 3-year process–managed by a research team led by IRCCS INRCA and supported by the DFP and national government–to sustain the policy design and recommendation in the AA area. A multilevel, co-managed coordination of AA policies was initiated by creating a stakeholder group with representatives from national and regional policy makers, civil society, and research community. A four-stage work plan was launched for addressing all phases of policy cycle (agenda setting, policy formulation, decision-making, implementation and monitoring) and support both national and regional policy makers in improving and designing new AA policies.

The 4-phase model, on which the PoA is based, represents a relevant innovation not only for the Italian context, but more in general as first step for those countries which need to plan a national AA strategy. By promoting new AA policies, the process contributes to develop new opportunities for social inclusion and offer older people with different profiles (e.g., health, socio-economic status) new patterns for being engaged in local communities and society. The mechanism initiated by the PoA constitutes both a social innovation [56,57] and a policy innovation [37,38] for AA, since it stimulates a multilevel governance and consultation with a heterogenous stakeholder group which would had never occurred otherwise. The interaction amongst stakeholders is essential to develop common ground, policy objectives and measures for tackling the AA challenge in a comprehensive and coordinated way.

While the evidence illustrated here represents the first systematic description of the innovative policy developments taking place in this field in Italy, this presentation suffers from some limitations, the main one being the absence of primary data collection and analysis. In fact, the overarching goal of this paper is to share knowledge about the PoA as a policy innovation on AA in Italy, which is not yet well known at international level, so that it may inspire and/or connect with initiatives carried out by other governments, researchers and international organisations. The lack of primary data presentation is obviated, however, by a detailed overview of the methods, activities and expected impacts of the ongoing PoA. This outline focuses not only on how researchers can both analyse AA policies and proactively lead and contribute to shape policy design and recommendation throughout all stages of the policy cycle, which is not a usual practice in the field.

The work on the PoA was initiated in the second half of 2019, and currently (Autumn 2020), the project is transiting from the stage of agenda setting (Task 1) to policy formulation (Task 2). First results became recently available, with the publication of a national state-of-the-art report [50] and over 30 individual reports on regional or sectorial AA policies [43]. In parallel to the progression of PoA tasks, the core team will focus on the in-depth analysis and evaluation of such policies, not only for domestic stakeholders but also for international research and policy communities. In particular, future research directions based on PoA results will have to investigate, among the others, the following aspects: the success factors and barriers of designing and implementing effective AA policies; what are the main gaps in current policies and how they can be addressed, on the basis of other national experiences; how AA policies and public debates evolve over time (at national and regional level) and what effects they have; how policies may balance the rights of older people to well-being, social inclusion and AA and the protection of individual and community health during public health emergencies (like the COVID-19 pandemic).

Although the PoA could not produce yet a clear policy impact, it undeniably represents a large, ambitious and systematic attempt to guide policy design and development on the basis of two pillars: an EBPM approach, relying on a detailed policy analysis throughout the policy cycle, and a continuous stakeholder involvement, oriented to address individual contexts of regions and specific social needs of the target group.

The PoA presents promising developments but also clear challenges, and they will also have to be tackled by future research analyses. First, the process relies especially on the last phase of policy implementation of the work planned and conducted for the PoA. This is due to the fact that the process will involve politicians, managers and officers at different levels, and that network of stakeholders engaged in it is large and will certainly require constant efforts to maintain it active, keeping all relevant actors involved in the loop. Second, a time span occurs between the PoA and the definition of the IAAS. Possible difficulties and delays could occur in recruiting, committing and retaining regional and national representatives in such a long process in this under-investigated policy area and in this particular period affected by the pandemic issues. Third, the Italian welfare system is still based on a conceptualization of the life-course as composed of strictly separated segments, identified only on the basis of age and without clear references to older people as active citizens. This is due to a clear division in social policies between the working population and retirees, typical of the Fordist age. A cultural change is therefore needed at both policy and societal level in order to overcome the current framework and the related barriers.

Furthermore, the PoA was designed and launched in 2019, before the COVID-19 pandemic started and brought substantial changes in citizens’ lifestyles and social policies for addressing the public health emergency in Italy, as in most of countries worldwide. In this context, the overall structure of the PoA was not altered, since it still reflects the traditional policy cycle and necessary steps for policy design and recommendation. However, it is clear that population needs and priorities have partially changed in relation to older people and AA, and this fact requires a further reflection within the stakeholder group in order to adjust task aims, guidelines and future policies accordingly. Such adjustment work will continue throughout the process, both within the stakeholder group and among the team of researchers, and will benefit also from discussion and guidance at international level [58].

More in general, a crucial role also lies on the research team capacity to show clearly the possible benefits of the PoA for all stakeholders, as well as to support and motivate them in defining and planning the achievement of their goals. The ambition remains to stimulate Regions/APs, as well as national institutions and policy makers, to improve the attention on AA activities, following recognized common principles and (national and international) good practices. This shall not, however, affect autonomy and independence of Regions/APs in relation to matters subject to their authority. The integrated role of DFP and IRCCS INRCA, as well as that of the Ministry of Labour and Social Policies and INAPP, as public advisors, represents in this regard an added-value opportunity for regional institutions to find all necessary support through their multidisciplinary and inter-institutional expertise, and not as an attempt to overcome local competences and rightful decision-making processes. The participatory approach adopted aimed to guarantee interest and perceived advantages to stakeholders, including representatives of Regions/APs in charge of implementing regional policies.

## 5. Conclusions

The current PoA and the future IAAS aspire to ensure a comprehensive understanding and management in terms of interventions, about how the different facets composing AA can be practically considered, developed and improved in the medium- and long-term in Italy. In fact, in the light of the lessons learned from international experiences, the process aims to stimulate connections between public, non-profit and research stakeholders at different levels, in order to increase cross-sectoral awareness among different policy areas linked to social participation and inclusion in old age. In addition, the participatory approach used is a fundamental key driver for enabling a wide participation, contribution and co-design of all concerned stakeholders in an inclusive way. This approach supports the prevention of different risks, like imposing unsuccessfully top-down methods, guidelines and intervention plan, and proposing recommendations not tailored to regional needs and local contexts.

Ultimately, the PoA aims to influence and promote a cultural change amongst national institutions (e.g., Ministries), Regions/APs and stakeholders, leading to the introduction of new and better policy actions for older people and AA. The role of older people in communities and society shall be reframed and empowered by policies promoting social, economic and cultural opportunities for AA. Furthermore, the increasing wealth, changing attitudes, motivations and lifestyles of old-age groups are leading to new ways to satisfy individuals’ social, family, individual and consumption needs (e.g., “silver economy”), to be adequately considered in a renewed policy framework on AA.

In the long-term, the general expectation, also raised clearly by stakeholders involved in the process so far, is that the current PoA could stimulate the national government to design a new national framework law on AA. This would constitute a starting point, a milestone for guaranteeing common principles and recommendations, as well as the allocation of dedicated resources, to public and private actors, in order to enhance policies, practices and services for a better participation and contribution of older people to the society.

The further challenge imposed by the COVID-19 emergency will guide the future implementation of the PoA and development of the IAAS. The COVID-19 pandemic, initiated in 2020 and caused by the spreading of coronavirus SARS-CoV-2, has massively impacted older people in relation to their health status, psychological well-being, social inclusion, access to services and social resources in general. Older people are at higher risk of infection from COVID-19 and at higher risk of death in comparison to younger generations. Furthermore, policy responses for limiting the spreading of COVID-19 included measures like “social” (or, better, “physical”) distancing and community/city lockdowns, which totally altered lifestyles of older people and general populations [28]. Therefore, the COVID-19 pandemic recently led to new barriers and challenges for AA, for which some social activities and opportunities are still limited for older people in many European countries.

A better and more integrated contribution of AA to healthy ageing—thus considering the new aim of health prevention from COVID-19—shall be thus deepened. In general terms, there is need to create a new convergence between AA and health policies (as well as with all other policy fields), by understanding how to guarantee AA opportunities in social contexts and physical environments where personal safety could be at risk. As recently highlighted by the pandemic, a rigid generational division of social relations and participation further contributes to create and widen breaches in the welfare system. This forces the different social groups to select their way of performing self-determination and putting frailest groups more and more at risk of social exclusion. A holistic vision of old age in times of pandemic shall therefore be explored and proposed under the framework of intergenerational solidarity, for better living and social participation of all individuals in the society. Therefore, it becomes extremely important to understand how an AA comprehensive and multidimensional approach might be still developed to have an impact on the population, considering the ongoing socio-cultural evolution of older population as well as the social changes imposed in the short- and medium-term by the COVID-19 pandemic.

## Figures and Tables

**Table 1:** List of stakeholders engaged in the PoA.

GROUP A—NATIONAL GOVERNMENT AND NATIONAL PUBLIC INSTITUTIONS			
Ministry of Economic Development	Ministry of Economy and Finances	Ministry of Environment	Ministry of Education
Ministry of Foreign Affairs and International Co-operation	Ministry of Internal Affairs	Ministry of Labour and Social Policies	Ministry of University and Research
Governmental Office for Sport	Interministerial Committee for Human Rights (CIDU)	Interregional Centre for Information, Statistical and Geographical Systems (CISIS)	National Association of Italian Municipalities (ANCI)
National Institute of Health (ISS)	National Institute for Insurance against Accidents at Work (INAIL)	National Institute for Social Protection (INPS)	National Institute of Statistics (ISTAT)
National Institute for Public Policy Analysis (INAPP)			
**GROUP B—REGIONAL GOVERNMENTS**			
***North-West***			
Piedmont	Valle d’Aosta	Liguria	Lombardy
***North-East***			
AP of Bolzano	AP of Trento	Veneto	Friuli-Venezia Giulia
Emilia-Romagna			
***Centre***			
Tuscany	Umbria	Marche	Latium
***South***			
Abruzzo	Molise	Campania	Apulia
Basilicata	Calabria		
***Islands***			
Sicily	Sardinia		
**GROUP C—OTHER NATIONAL ORGANISATIONS AND ACADEMIA**			
***Non-profit organizations, unions and professional associations***			
50&Più Confcommercio	Age Platform Italia	Anap Confartigianato	Ancescao
Anpa Confagricoltura	Anpecomit	Anziani e non solo	Associazione Lavoro Over 40
Associazione Nazionale Tutte le Età Attive per la Solidarietà (ANTEAS)	Associazione Pensionati CIA	Associazione per i Diritti degli Anziani (ADA)	ATDAL Over 40-Associazione nazionale tutela diritti over 40
Atdal over40	AUSER	Cia Pensionati	Coordinamento Unitario dei Pensionati del Lavoro Autonomo (CUPLA)
Cna Pensionati	Fap Acli	Fap Credito	Federpensionati Coldiretti
Fipac Confesercenti	FNPA Casartigiani	FNP-CISL	Fondazione Nazionale Assistenti Sociali
Forum delle Associazioni Familiari	Forum Terzo Settore	Over 50 della Confeuro	S.a.pens. Or.s.a
Sindacato Pensionati Confagricoltura	Società italiana di gerontologia e geriatria (SIGG)	Solimai	SPI-CGIL
UGL Pensionati	UILP-UIL	Unitre	UNPLI-Unione Proloco NAZIONALE
***Academic and research stakeholders***			
Institute for Research on Population and Social Policies (IRPSS), National Institute for Research (CNR)	La Sapienza University, Rome	Catholic University of the Sacred Heart, Milan	University of Palermo
University of Tor Vergata, Rome			
